# Integrating machine learning algorithms to systematically assess reactive oxygen species levels to aid prognosis and novel treatments for triple -negative breast cancer patients

**DOI:** 10.3389/fimmu.2023.1196054

**Published:** 2023-06-19

**Authors:** Juan Li, Yu Liang, Xiaochen Zhao, Chihua Wu

**Affiliations:** ^1^ Department of Breast Surgery, Sichuan Provincial People’s Hospital, University of Electronic Science and Technology of China, Chengdu, China; ^2^ Chinese Academy of Sciences Sichuan Translational Medicine Research Hospital, Chengdu, China; ^3^ Department of Hepatobiliary Surgery, Sichuan Provincial People’s Hospital, University of Electronic Science and Technology of China, Chengdu, China

**Keywords:** triple negative breast cancer (TNBC), reactive oxygen species (ROS), machine-learning, chemotherapy, immunotherapy

## Abstract

**Introduction:**

Breast cancer has become one of the top health concerns for women, and triple-negative breast cancer (TNBC) leads to treatment resistance and poor prognosis due to its high degree of heterogeneity and malignancy. Reactive oxygen species (ROS) have been found to play a dual role in tumors, and modulating ROS levels may provide new insights into prognosis and tumor treatment.

**Methods:**

This study attempted to establish a robust and valid ROS signature (ROSig) to aid in assessing ROS levels. The driver ROS prognostic indicators were searched based on univariate Cox regression. A well-established pipeline integrating 9 machine learning algorithms was used to generate the ROSig. Subsequently, the heterogeneity of different ROSig levels was resolved in terms of cellular communication crosstalk, biological pathways, immune microenvironment, genomic variation, and response to chemotherapy and immunotherapy. In addition, the effect of the core ROS regulator HSF1 on TNBC cell proliferation was detected by cell counting kit-8 and transwell assays.

**Results:**

A total of 24 prognostic ROS indicators were detected. A combination of the Coxboost+ Survival Support Vector Machine (survival-SVM) algorithm was chosen to generate ROSig. ROSig proved to be the superior risk predictor for TNBC. Cellular assays show that knockdown of HSF1 can reduce the proliferation and invasion of TNBC cells. The individual risk stratification based on ROSig showed good predictive accuracy. High ROSig was identified to be associated with higher cell replication activity, stronger tumor heterogeneity, and an immunosuppressive microenvironment. In contrast, low ROSig indicated a more abundant cellular matrix and more active immune signaling. Low ROSig has a higher tumor mutation load and copy number load. Finally, we found that low ROSig patients were more sensitive to doxorubicin and immunotherapy.

**Conclusion:**

In this study, we developed a robust and effective ROSig model that can be used as a reliable indicator for prognosis and treatment decisions in TNBC patients. This ROSig also allows a simple assessment of TNBC heterogeneity in terms of biological function, immune microenvironment, and genomic variation.

## Introduction

The incidence of breast cancer (BC) has gradually increased in recent years and has been recognized as one of the most frequently diagnosed types of cancer (1, 2). BC is a type of tumor that is highly heterogeneous, with major molecular subtypes defined according to different hormone receptor expressions (estrogen receptor (ER), progesterone receptor (PR), and human epidermal growth factor receptor 2 (HER2)) (3). Triple-negative breast cancer (TNBC), which does not overexpress HER2 and is devoid of hormone receptor expression (ER/PR), is an invasive subtype that usually exhibits extensive intratumoral heterogeneity (3). Due to the extensive intratumoral heterogeneity, TNBC lacks effective biomarkers and poses a difficult challenge for targeted therapy, with patients often experiencing treatment resistance (4). In clinical practice, TNBC has a high rate of early recurrence and is more susceptible to metastasis, making it the BC subtype with the poorest prognosis (5). Despite emerging research that has made multiple advances in elucidating the mechanisms of tumor progression, the clinical outcomes of TNBC remain worrisome. This grim fact underscores the urgent need to find reliable biomarkers for TNBC and to develop novel, effective treatments.

Reactive oxygen species (ROS) are byproducts of various aerobic metabolic pathways and are more reactive but less long-lived than common oxygen molecules (6). Excessive ROS enrichment has been detected in various cancers, and a dual role of ROS in the cancer process has been recognized (7). On the one hand, ROS can activate protumorigenic signaling, regulate cancer cell proliferation and differentiation, and drive DNA damage and chromosomal instability in the nucleus (6). This set of modulations undoubtedly increases intratumor heterogeneity. On the other hand, ROS can be involved in multiple cell death pathways by way of oxidative stress and induce apoptosis in tumor cells (8). In contrast to normal cells, abnormal oxidation−reduction homeostasis is maintained within tumor cells, sustaining activation of pro-tumor pathways and anti-apoptosis (8). In light of these findings, targeting the ROS pathway has emerged as a new direction in the treatment of cancer, where modulating tumor cell ROS levels can induce apoptosis and increase sensitivity to chemotherapeutic agents (9, 10). In addition, new studies have focused on immune cells in the microenvironment, proposing that ROS can regulate the activity and function of a variety of immune cells (11, 12). For example, excess ROS can act as a potential antigenic stimulus to convene dendritic cells and T cells in the microenvironment and increase their infiltration levels, thus effectively increasing antitumor responses (13, 14). In addition, it has also been found that regulation of ROS concentration can reduce the secretion of immunosuppressive cytokines and decrease the summoning of suppressive immune cells, thus avoiding immune escape (15). Taken together, ROS may also function as an effective target for immunotherapy. In recent years, targeted therapies and immunotherapy have become effective complements to conventional chemotherapy and have amazing potential in improving the prognosis of TNBC patients. However, further research is still needed on how to accurately modulate ROS to increase targeted therapy and immunotherapy, and the development of effective biomarkers is urgently needed.

In this study, we integrated sequencing data from TCGA and Metabric to systematically analyze the ROS regulatory pathways in TNBC and identified 24 potential ROS regulatory factors. An optimized bioindicator ROS signature (ROSig) was developed through an integrated machine learning pipeline. The prognostic significance, biological and immunological heterogeneity, and clinical application potential of ROSig were subsequently evaluated in detail. Furthermore, our findings were confirmed by single-cell sequencing data and cell counting kit-8 (CCK8) experiments. Our study reveals the possibility of ROS as a novel TNBC bioindicator, providing new insights into the prognosis and combination treatment options for TNBC patients.

## Methods

### Data acquisition and preprocessing

We retrieved BC data from TCGA using the UCSC Xena platform (https://xena.ucsc.edu/), selected samples with a pathological diagnosis of TNBC, and enrolled a total of 195 patients as a TCGA-TNBC cohort after excluding patients with missing clinical information (follow-up, staging, age). The corresponding RNA-seq, maf mutation data, and copy number variation (CNV) data processed by Gistic 2.0 were downloaded. Subsequently, transcriptional profiles and patient information for the Breast-Metabric cohort were downloaded via the cBioPortal platform (http://www.cbioportal.org/), a dataset that included a total of 418 TNBC patients and was used as an external validation cohort (16).

To assess the applicability of ROSig for immunotherapy prediction, we downloaded RNA-seq data from two well-established immunotherapy cohorts containing clinical follow-up data as well as detailed records of the number of mutations and neoantigens: a. the Imvigor210 cohort containing 298 patients with bladder cancer who received anti-PD-L1 therapy; b. the 121-patient melanoma Liu David cohort, where patients received anti-PD-1 therapy (17, 18). All RNA-Seq data were log2 normalized and z scored using the scale function.

Finally, we downloaded the single-cell transcriptome GSE176078, a dataset containing 10 primary TNBC samples with a total of 42,112 cells. The “seruat” package was used for normalization and cell clustering according to the original parameters (19). Specifically, we retained cells with >200 expressed genes and <20% mitochondrial gene content. Using the default parameter “NormalizeData” to normalize the expression profile, 2000 feature genes were selected for dimensionality reduction. Adjacent modules were identified based on 30 principal components and a resolution of 0.8, and the cell types were identified according to the original annotation file (19).

### Machine learning -based system pipeline for generating ROSig

We collected ROS-related pathways from the ontology gene set in the MSIGDB (https://www.gsea-msigdb.org/gsea/msigdb) database, containing a total of 406 ROS-related genes in 15 pathways (20). A detailed list of ROS pathways is provided in **Table S1**. To systematically and efficiently retrieve the best combination of machine learning models to generate the most reliable ROSig. We performed the following pipeline: a. Single-factor Cox regression to retrieve indicators with significant prognostic efficacy in ROS-regulated pathways (p<0.05); b. Integration of nine well-established machine-learning algorithms, including CoxBoost, stepwise Cox, Supervised Principal Component (SuperPC), Elastic Network (Enet), generalized augmented regression model (GBM), Random Survival Forest (RSF), Survival Support Vector Machine (survival-SVM), Lasso-penalty Cox regression (LASSO), and Ridge. A combination of two algorithms, one for filtering variables and the other for constructing the model, was composed, resulting in the final 63 combinations of algorithms (21). The default parameters were applied, and five cross-validations were performed to avoid overfitting. c. The constructed models were used in the Metabric queue to verify the performance, and the best model was selected by the C-index, with a higher C-index indicating a more accurate model (22). ROSig for different datasets was generated using the best model by the predict function, and high and low ROSig patients were classified according to the median of ROSig.

### Detection of cell proliferation

We purchased two TNBC cell lines (MDA-MB-231 and BT549) from Shanghai EK Bioscience Co. We then transfected the cells using LipofectamineTM 2000 transfection reagent (Invitrogen, USA) according to the instructions to silence the target genes and set up a blank control. CCK-8 (Bioss, China) was used to measure the proliferation rate of TNBC cell lines. Three chambers of different groups were selected at 0, 12, 24, 48, and 72 hours. Subsequently, 10 ml of CCK-8 was added and incubated at 37°C for two hours according to the instructions. The absorbance value at 450 nm was measured to estimate the proliferation rate. All cells were grown in DMEM containing 10% FBS and incubated in a cell culture incubator at 37°C with 5% CO_2_.

### Detection of cell invasion

After transfected TNBC cells were cultured for 48 hours, they were inoculated into the upper chamber of a Transwell plate coated with Matrigel solution (BD Biocoat, USA). After incubation in the incubator for 48 h, the cells were fixed with 4% paraformaldehyde and stained with 0.1% crystalline violet. The invasion level of cells was observed by light microscopy, and cells were counted by ImageJ software.

### Dissecting the heterogeneity of biological functions and the immune microenvironment

Differentially expressed genes (DEGs) from different ROSig subgroups were screened using the “limma” software package with a threshold of fold change>2 and adjusted p value<0.05 and functionally annotated and enriched through the Metascape website (https://metascape.org/gp). Subsequently, the enriched KEGG pathway was assessed by GSEA software (version 4.1.0) for different ROSig subgroups. The relative activity of the HALLMARK gene set was assessed using the ssGSEA algorithm based on the “gsva” package. In addition, markers for antitumor immune circulation were collected according to previous definitions (23). We then used the “CIBERSORT” algorithm to estimate the relative infiltration abundance of 22 immune cells in the microenvironment based on transcriptional profiles (24). The individual patient’s Estimate score was also estimated by the “ESTIMATE” algorithm (25). The homologous recombination defect (HRD) score, intratumor heterogeneity, indel neoantigens, and SNV neoantigens of TCGA-TNBC patients were retrieved from the previous literature (26). The “Nebulosa” package was used to display the density of ROSig in different cells (27). Finally, the “CellChat” package was used in the single-cell dataset to identify possible crosstalk between different cells in the tumor microenvironment of different ROSig subgroups (28).

### Dissecting the heterogeneity of genomic variants

For the maf mutation data, we used the “maftools” package for processing and analysis (29). The total number of nonsynonymous mutations in individual samples was first calculated, and then high frequency mutated genes were identified based on a threshold of mutation frequency >5, and differences in mutation frequency between ROSig groups were assessed. Chromosomal amplifications and deletions in CNV data were identified according to a threshold of 0.2. “Complexheatmap” was used to present CNV profiles of different ROSig groups. The total number of amplifications and deletions of individual samples were counted and presented with the ggplot2 package.

### Assessing the potential of ROSig for clinical application

We evaluated the predictive potential of ROSig for chemotherapy, targeted therapies, and immunotherapy. First, the IC50 values of the samples for chemotherapeutic agents were predicted using the ridge regression function in the “pRRophetic” package based on the GDSC database (version 2016.) (30). Subsequently, the sensitivity of different ROSig patients to immunotherapy was assessed by the TIDE algorithm (http://tide.dfci.harvard.edu). We uploaded the top 150 differentially expressed genes to the Cmap database (https://clue.io/) to predict the potential small molecule agents that may target ROS. Finally, the predicted sensitivity to immunotherapy was assessed by ROSig generated in two established immunotherapy cohorts (Imvigor210 and Liu David).

### Statistical analysis

All statistics and plots were performed in the R environment (version 4.1.0). For the comparison of two groups, Student’s t test or Wilcoxon’s rank test was chosen according to the data structure. Fisher’s exact test was used for the comparison of rank data. The log-rank test was used to detect differences between survival curves. Correlation analysis was performed by the Pearson coefficient. Two-tailed P<0.05 was set as the threshold of significance if not otherwise stated.

## Results

### Dissecting the transcriptome features of ROS-regulated genes in TCGA-TNBC

**Figure 1:**
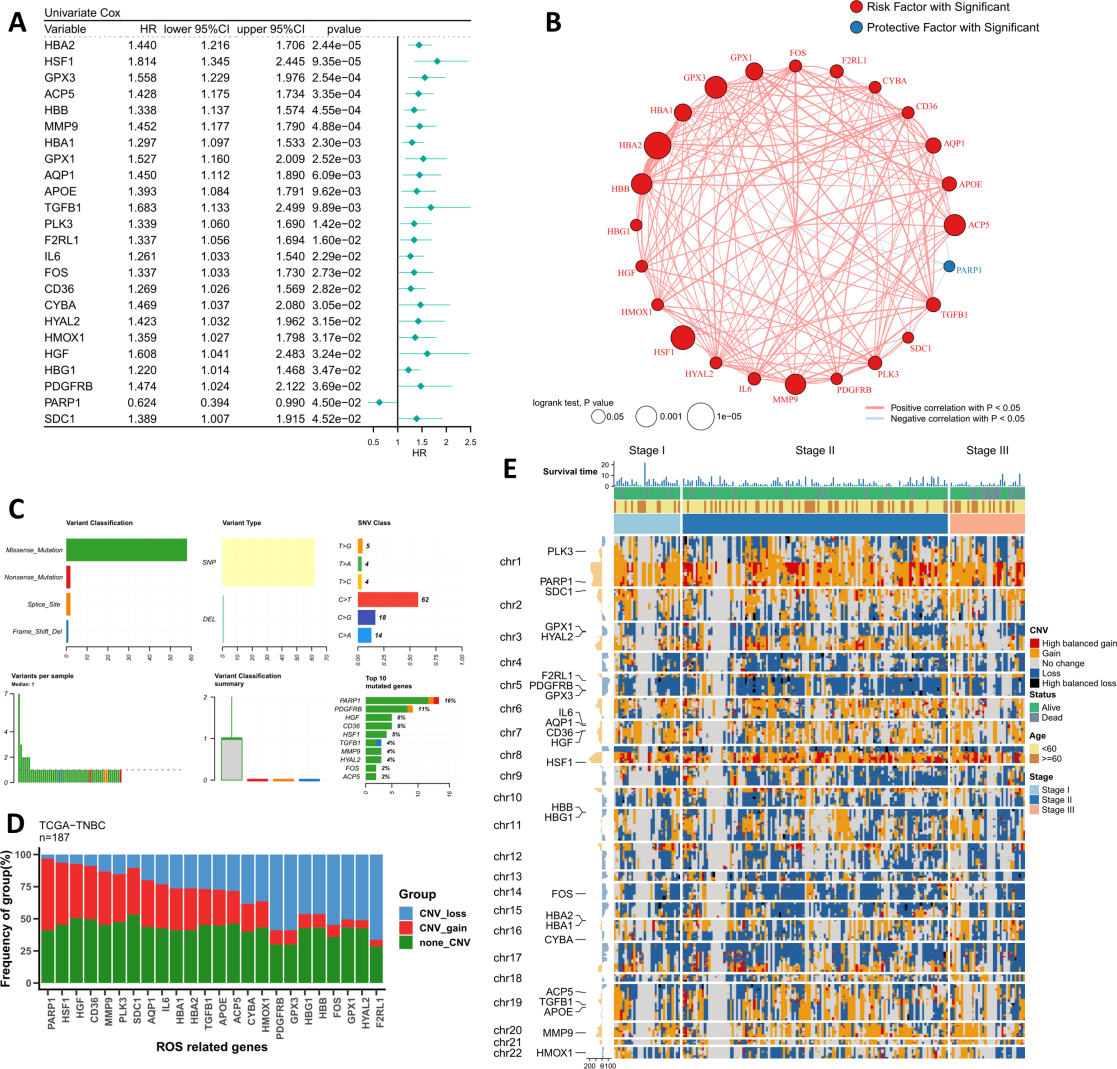
Genomic profiling of the driving ROS indicators. **(A)** The forest plot shows the results of the univariate Cox regression for the 24 ROS indicators. **(B)** The correlation network of 24 ROS indicators in TCGA-TNBC. **(C)** Summary of single nucleotide variants of 24 ROS indicators in TCGA-TNBC. **(D)** Summary of copy number variants of 24 ROS indicators in TCGA-TNBC. **(E)** The landscape of copy number variants of 24 ROS indicators in different stage patients.

We first searched for indicators of independent prognostic efficacy in the ROS regulatory pathway and finally identified 24 significant regulators (**Figure 1A**). Among them, HBA2 and HSF1 were the two most significant risk factors. Subsequently, we mapped the correlation network of these 24 ROS modifiers, and the results showed that all 23 indicators except PARP1 were highly positively correlated (**Figure 1B**). Interestingly, PARP1 was the only protective factor. We summarized their mutational landscapes, and the results showed that missense mutations accounted for the highest percentage and that PARP1 was the gene with the highest mutation frequency (**Figure 1C**). More interestingly, we found that PARP1 and HSF1 were the most frequently amplified genes, while F2RL1, GPX3, and PDGFRB were the three genes with the highest deletion frequency (**Figure 1D**). **Figure 1E** shows the CNV profiles of patients with different stages of TNBC in detail, and we can found that a higher proportion of TNBC patients in stage II in the entire dataset.

### Integrating machine learning pipeline to build a robust ROS signature

**Figure 2:**
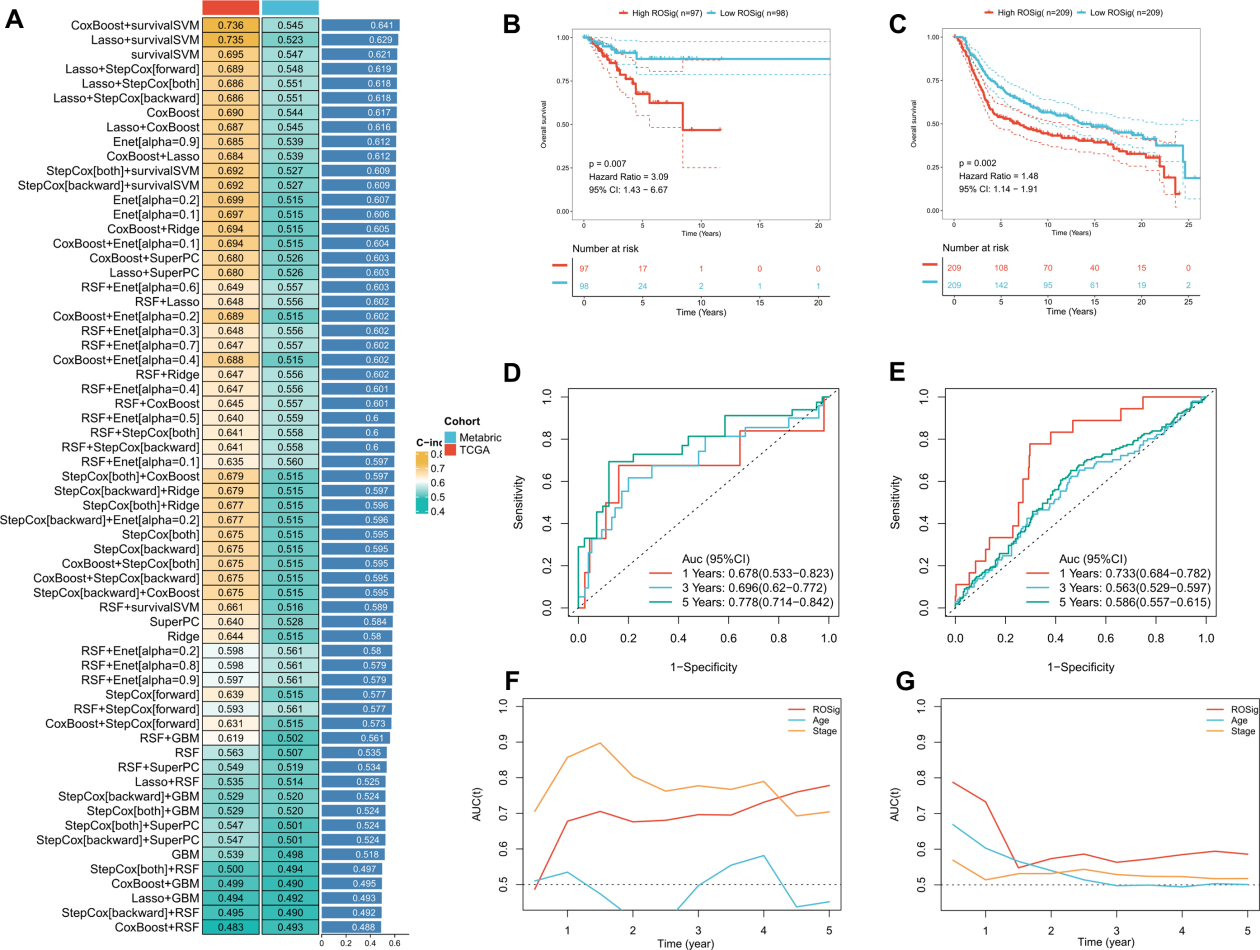
Systematic pipeline of integrated machine-learning algorithms to construct ROSig. **(A)** The c-index of a total of 61 algorithm combinations in both the TCGA and METABRIC cohorts. **(B)** KM survival curves for the high ROSig and low ROSig groups in the TCGA cohort. **(C)** KM survival curves for the high ROSig and low ROSig groups in the Metabric cohort. **(D)** 1-, 3-, and 5-year ROC curves for ROSig in the TCGA cohort. **(E)** 1-, 3-, and 5-year ROC curves for ROSig in the Metabric cohort. **(F)** TimeROC curves for ROSig and clinical characteristics in the TCGA cohort. **(G)** TimeROC curves for ROSig and clinical characteristics in the Metabric cohort.

As stated in the methods section, we imported 24 independently prognostic ROS modulators into the machine learning algorithm pipeline and performed a 5-fold cross-validation. Based on the average C-index, we found that Coxboost+survivalSVM was the best combination (C-index: 0.736 for TCGA; 0.545 for Metabric). Therefore, we applied this combination to generate ROSig (**Figure 2A**) in TCGA and Metabric queues. The results of the survival analysis indicate a statistically significant impact of risk stratification utilizing ROSig in both cohorts. Specifically, patients classified as having a high ROSig exhibited significantly worse survival outcomes. (**Figures 2B, C**). ROC analysis showed that ROSig was an excellent predictor in the TCGA cohort (**Figure 2D**). In contrast, in the Metabric cohort, ROSig was superior at 1 year but poor at 3 and 5 years (**Figure 2E**). TROC compared the predictive merits of ROSig with age and stage metrics. In the TCGA cohort, ROSig outperformed Stage in predictive efficacy at 4 years with increasing time (**Figure 2F**), whereas in the Metabric cohort, ROSig had better predictive performance than Stage over a five-year period. (**Figure 2G**).

### Knockdown of HSF1 inhibits the proliferation and invasion of breast cancer cells *in vitro*

**Figure 3:**
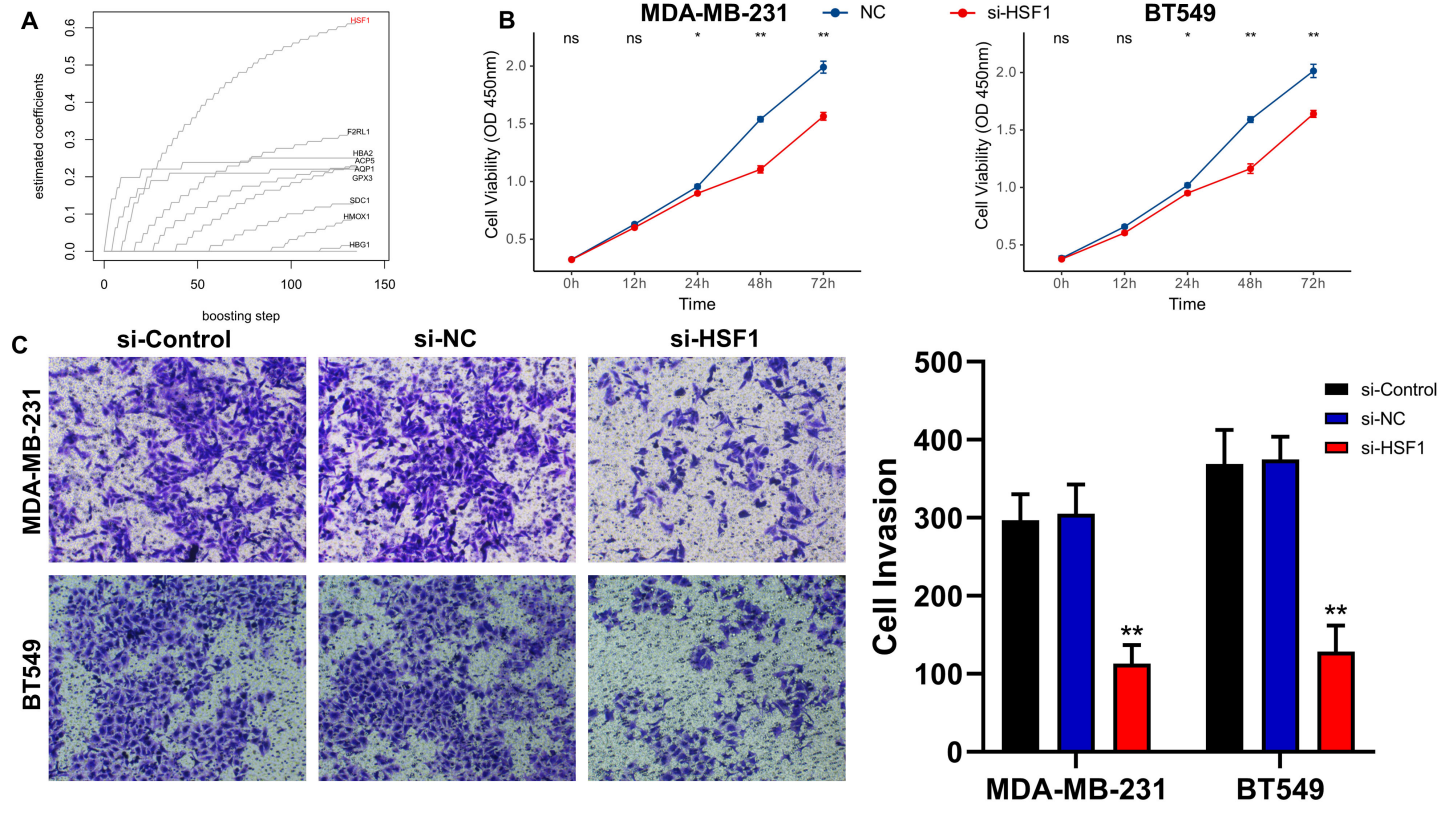
Knockdown of HSF1 expression affects the proliferation and invasion of breast cancer cell lines. **(A)** Using the Coxboost algorithm to filter the 9 best ROS indicators, HSF1 was identified as the core ROS gene. **(B)** The line graph shows the proliferation levels of different TNBC cell lines after knockdown of HSF1. **(C)** Images and statistical analysis of the transwell assay of TNBC cell lines after knockdown of HSF1. *P<0.05, **P<0.01.

We then examined the effect of core genes on the malignant phenotype of BC in the ROSig model *in vitro*. Specifically, Coxboost was used as a screening algorithm to select the 9 best indicators, with HSF1 having the leading edge (**Figure 3A**). To verify the promoting effect of HSF1 on tumor progression, we performed a CCK-8 assay in two BC cell lines (MDA-MB-231 and BT549), and the results showed a significant downward trend in the cell proliferation level after knockdown of HSF1 (**Figure 3B**). Transwell assays showed that the number of invasive BC cell lines transfected with si-HSF1 was significantly lower than that of BC cell lines transfected with si-NC (**Figure 3C**). In summary, HSF1 can promote the proliferation and invasion of BC cell lines *in vitro.*

### Systematic evaluation of the predictive benefit of ROSig

**Figure 4:**
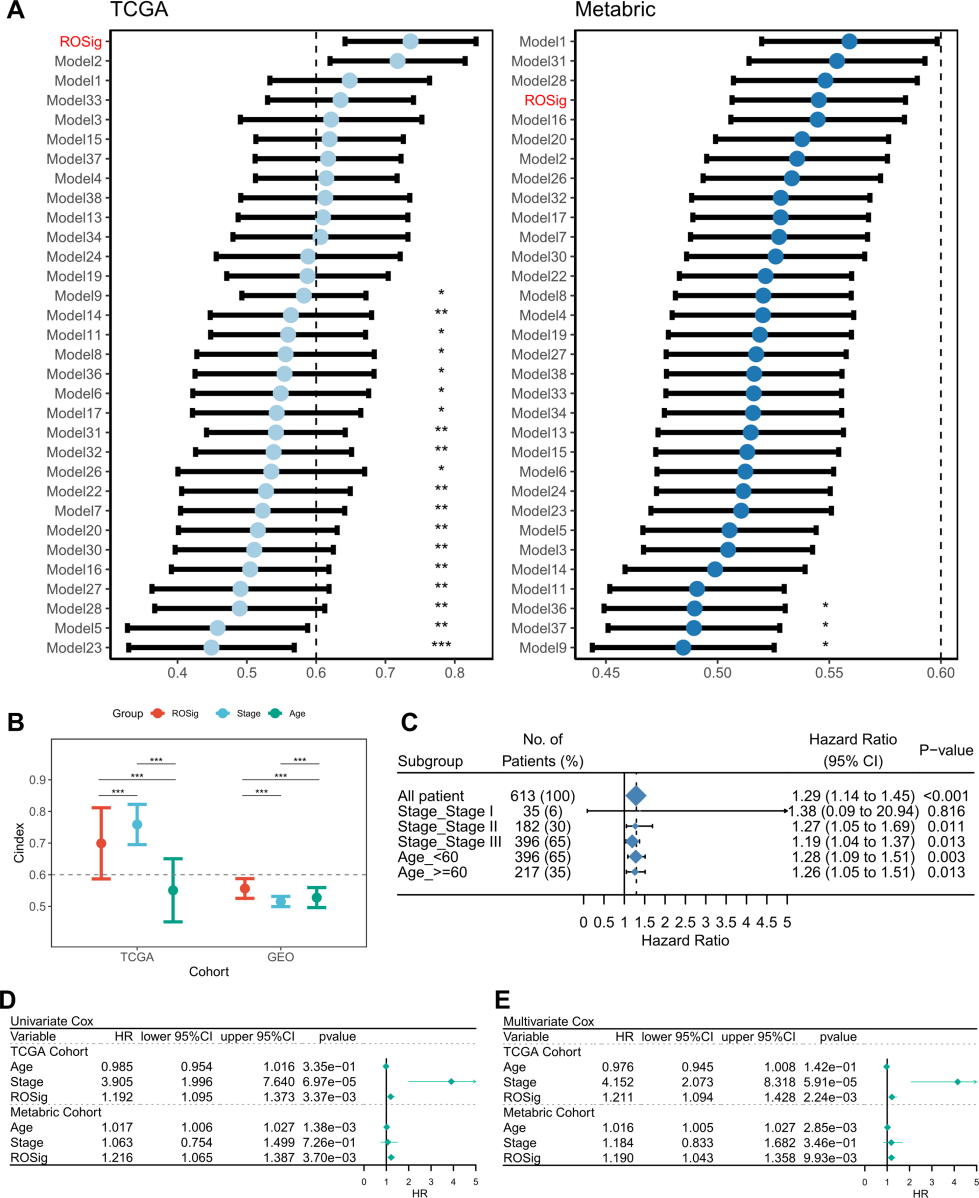
Evaluation of the ROSig model. **(A)** Comparing the accuracy of ROSig with 31 published molecular signatures for TNBC. **(B)** C-index for ROSig and clinical characteristics in both cohorts. **(C)** Subgroup analysis of ROSig. **(D)** Univariate Cox regression analysis of OS in TCGA and meta-GEO cohorts. **(E)** Multivariate Cox regression analysis of OS in TCGA and meta-GEO cohorts. *P<0.05, **P<0.01, ***P<0.001.

We searched for published gene signatures used to predict the prognosis of TNBC and collected a total of 38 prediction models based on RNA transcriptional profiles. The detailed gene signature was provided in **Table S2**. We excluded models with <3 valid genes at the time of model application and finally compared the advantages of 31 published models with ROSig. The results showed that ROSig was the best predictor in the TCGA cohort and had significantly higher predictive efficacy than 19 publicly available models (**Figure 4A**). In the Metabric cohort, ROSig was the fourth most effective metric and showed significant advantages in comparison with the three models (**Figure 4A**). Specifically, for our study, the C-index indicated that ROSig is an indicator with potential for clinical application (**Figure 4B**). Subgroup analysis indicated that ROSig performed poorly in predicting patients in the early stage (Stage 1) but had independent prognostic efficacy in all other subgroups (**Figure 4C**). More convincingly, both univariate and multifactorial Cox regression analyses confirmed ROSig as an independent prognostic indicator for both TNBC cohorts (**Figures 4D, E**).

### ROSig-based individual risk stratification

**Figure 5:**
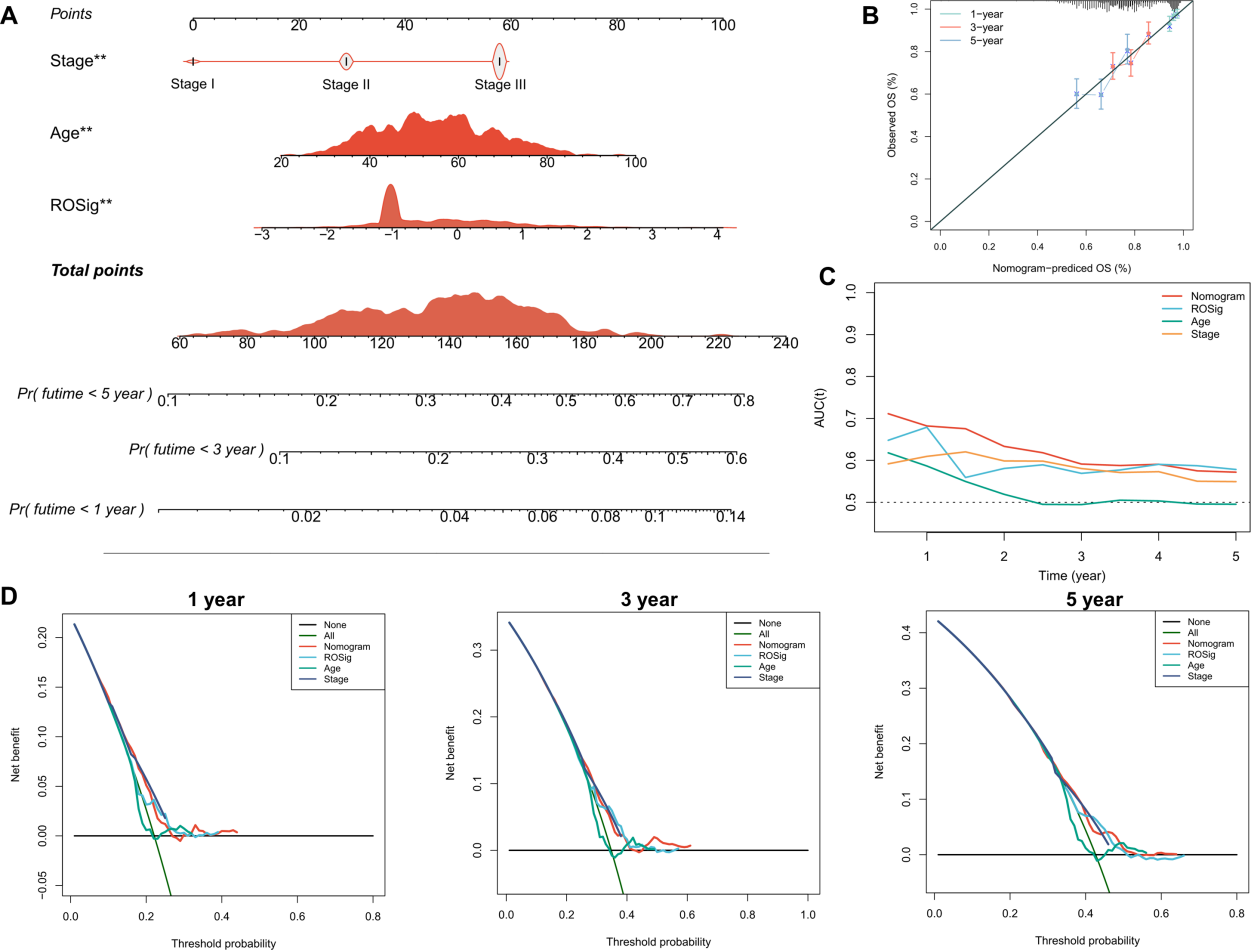
Individual risk stratification based on ROSig. **(A)** Constructing a nomogram using ROSig and clinical characteristics for risk stratification of individual patients. **(B)** The calibration curves for the nomogram at 1, 3, and 5 years. **(C)** TimeROC curves comparing the predictive accuracy of the nomogram and other clinical features. **(D)** 1-, 3-, and 5-year DCA curves for the nomogram and other clinical characteristics. **P<0.01.

To better facilitate the clinical application of ROSig, we integrated ROSig, age, and stage and developed a nomogram for rapid clinical application (**Figure 5A**). The calibration curve showed that the ROSig-based nomogram model showed good predictive performance at 1, 3, and 5 years (**Figure 5B**), and the TROC curve showed that the nomogram model was the best predictor over a 5-year cancer cycle (**Figure 5C**). More convincingly, the decision curve analysis (DCA) curve supports this conclusion, with the nomogram model having satisfactory decision gains at the 1-year, 3-year and 5-year points (**Figure 5D**).

### ROSig-based heterogeneity at single-cell resolution

**Figure 6:**
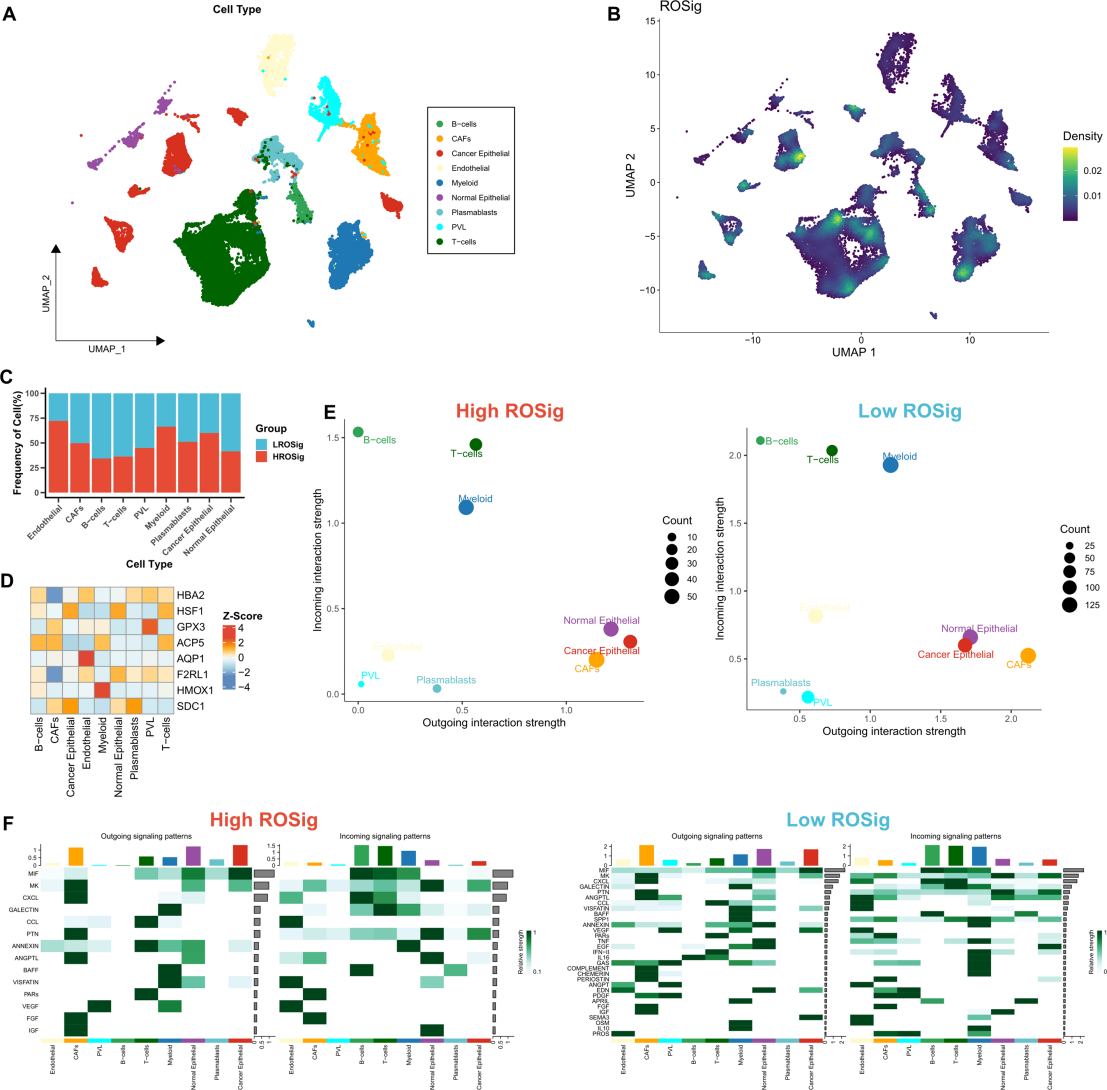
Dissecting cellular interactions of different ROSig groups at single-cell resolution. **(A)** Nine identified cell types are shown based on Umap descending. **(B)** Density of ROSig in different cell clusters. **(C)** Proportion of ROSig groups in different cell types. **(D)** Expression of nine indicators of the ROSig model in different cell subgroups. **(E)** Overall cellular communication intensity in cells with high ROSig (left) and low ROSig (right). **(F)** Specific communication pathways between cells with high ROSig (left) and low ROSig (right).

We then used single-cell datasets to resolve the heterogeneity of the microenvironment in different ROSig groups from more specific cellular interactions. We identified 9 cell subtypes based on the original parameters (**Figure 6A**). We then found a higher proportion of low ROSig cells in B and T cells and a higher proportion of high ROSig cells in cancer epithelial cells, myeloid cells, and endothelial cells (**Figure 6B**). Low ROSig cells were more predominant in B and T cells, while high ROSig cells were more abundant in cancer epithelial, myeloid, and endothelial cells (**Figure 6C**). The gene expression of the final ROSig model is also shown in **Figure 6D**, where HSF1 is expressed at higher levels not only in epithelial cells but also in T cells. We identified significant cellular exchange pairs based on a threshold of P<0.05, which showed that cells with low ROSig had more overall incoming and outgoing communication pairs (**Figure 6E**). **Figure 6F** shows detailed exchange pathways, there are fewer communicating pathways in high ROSig cells. In contrast, there are more communicating pathways in low ROSig cells, and most of them are related to the immune system (e.g., CXCL, TNF. IL16, etc.) (31–33).

### Dissecting the biological heterogeneity of different ROSig groups

**Figure 7:**
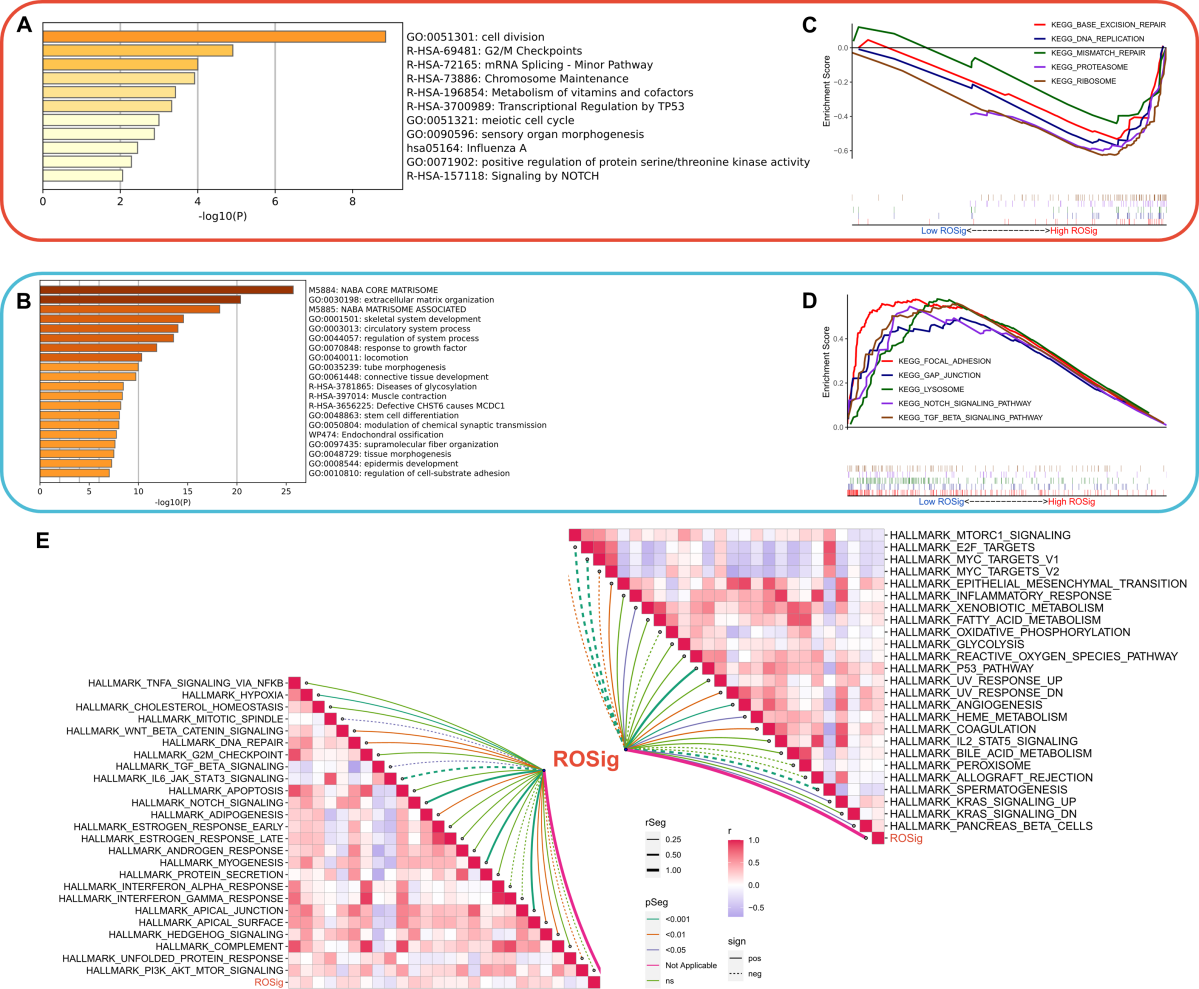
Dissecting the functional heterogeneity of ROSig. **(A)** Bar plot showing the biological pathways of upregulated gene enrichment in the high ROSig group. **(B)** Bar plot showing the biological pathways of upregulated gene enrichment in the low ROSig group. **(C)** GSEA revealed the top five enriched KEGG pathways in the high ROSig group. **(D)** GSEA revealed the top five enriched KEGG pathways in the low ROSig group. **(E)** The correlation network between ROSig and the activity of 50 hallmark pathways.

We then systematically assessed the potential biological functional heterogeneity behind different ROSig levels. First, the DEGs of different ROSig subgroups were functionally enriched. The results showed that DEGs in the high ROSig group were mainly involved in cell division, cell cycle-related pathways (including G2/M checkpoints, etc.) (**Figure 7A**). DEGs in the low ROSig group were mainly enriched in the extracellular matrix and cytoskeleton-related pathways (**Figure 7B**). GSEA showed that DEGs in the high ROSig group were mainly enriched in cell cycle-related pathways such as DNA replication, ribosome, and mismatch repair (**Figure 7C**). In contrast, the pathways upregulated in the low ROSig group were mainly the gap junction, lysosome, and TGF -β pathways (**Figure 7D**). Finally, we evaluated the correlation between ROSig and cancer marker pathways (HALLMARK set) and showed that ROSig was positively correlated with cell cycle-related pathways and metabolic-related pathways and negatively correlated with signaling pathways such as TGF-β, interferon, IL6-JAK-STAT3 and MYC (**Figure 7E**).

### Dissecting immune heterogeneity at different ROSig levels

**Figure 8:**
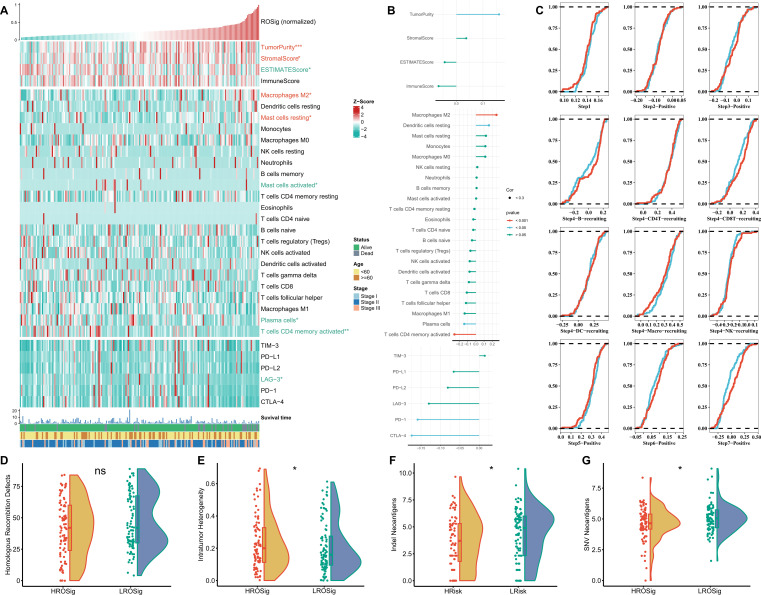
Dissecting the immune heterogeneity of ROSig. **(A)** Complex heatmap showing the ROSig landscape in the tumor immune microenvironment, including the ESTIMATE score, immune cell infiltration, and immune checkpoint expression. **(B)** The correlation between ROSig and immune indicators (including ESTIMATE score, immune cell infiltration, and immune checkpoint expression). **(C)** Cumulative distribution plots showing the difference in the anticancer immune cycle between different ROSig subgroups. Violin plot displaying the difference in **(D)** HRD score, **(E)** intratumor heterogeneity, **(F)** indel neoantigens, and **(G)** SNV neoantigens between different ROSig subgroups. *: P<0.05, ***: P<0.001, ns: not significant.

To analyze the balance of the immune microenvironment at different ROSig levels from multiple perspectives, we analyzed the relationship between Estimate score, immune cell infiltration abundance and checkpoint activity with ROSig. **Figure 8A** summarizes the immunological profile of ROSig. We found that high ROSig corresponded to higher tumor purity, stromal score, M2 macrophage abundance, and resting mast cell abundance. In contrast, the low ROSig group had a higher Estimate score, plasma cell and activated CD4-T-cell abundance, and higher LAG-3 expression (**Figure 8A**). In addition, we found that ROSig was significantly positively correlated with tumor purity, M2 macrophages, and resting dendritic cells (**Figure 8B**). Plasma cells, CD4-T cells, PD-1 and CTLA-4 were significantly negatively correlated with ROSig (**Figure 8B**). We then assessed the antitumor immune circulating activity in different ROSig groups, and the results showed that the low ROSig group was more active in Step 4 B-cell and CD8+ T-cell convening as well as in step 7 (**Figure 8C**). However, cloud and rain plots showed no significant difference in homologous chromosome recombination between the two ROSig subgroups (**Figure 8D**). However, tumor heterogeneity was greater in the high ROSig group (**Figure 8E**), and indel neoantigens and SNV neoantigens were more frequent in the low ROSig group (**Figures 8F, G**).

### Dissecting the potential genomic heterogeneity of ROSig

**Figure 9:**
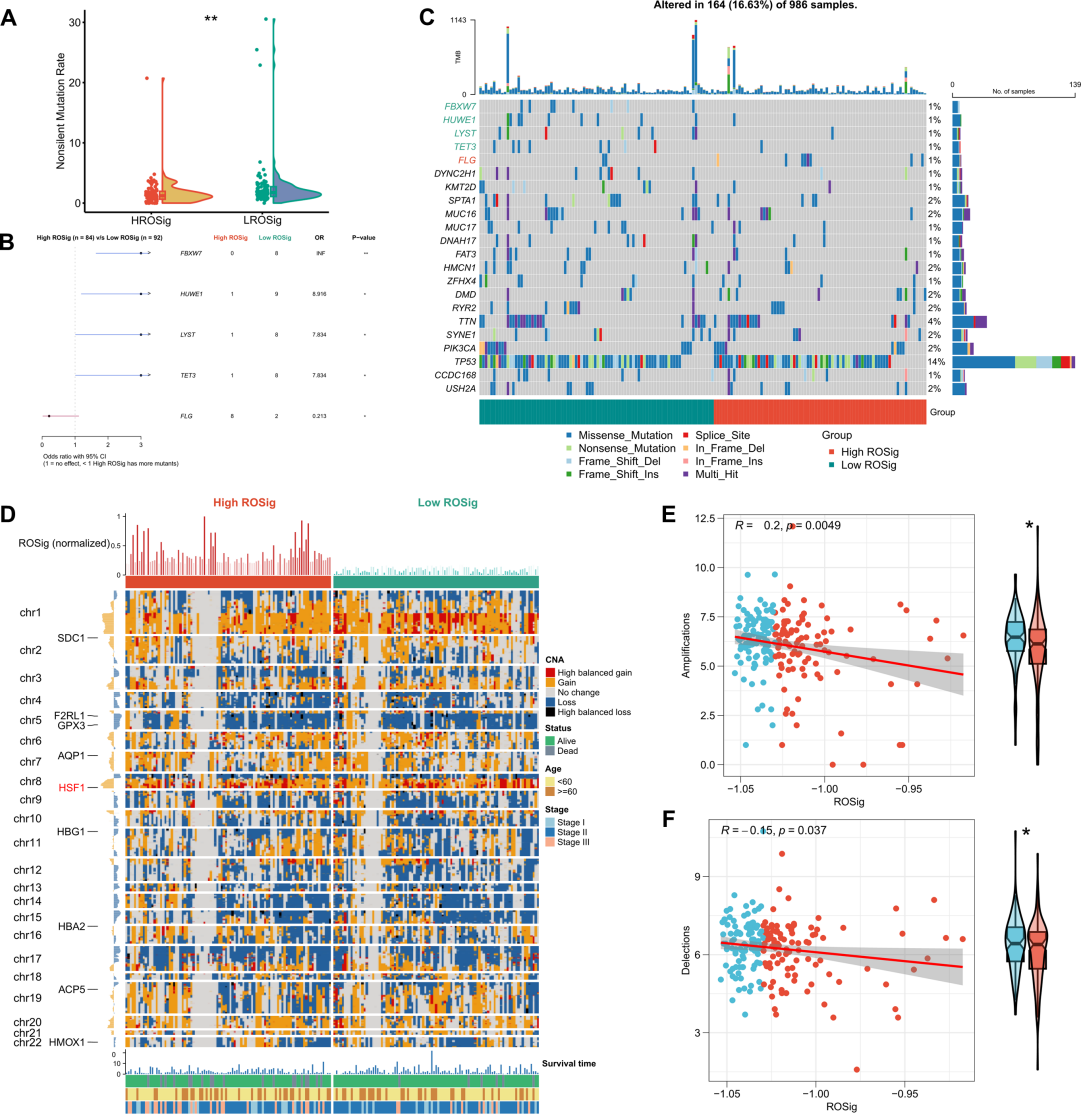
Dissecting the genomic mutational heterogeneity of ROSig. **(A)** Violin plot showing the difference in nonsynonymous mutations between different ROSig subgroups. **(B)** Forest plot showing statistically significant differences in high-frequency mutated genes between the high- and low-ROSig subgroups. **(C)** Waterfall plot of high-frequency mutated genes between the high- and low-ROSig subgroups. **(D)** Complex heatmap displaying the CNV landscape between high- and low-ROSig subgroups. Box plots and scatter plots show the correlation between ROSig and **(E)** Amplifications and **(F)** Delections. *P<0.05, **P<0.01.

We resolved the genomic heterogeneity of different ROSig levels from the perspective of single nucleotide mutations and CNV. First, the Rainy plot showed that the low ROSig group had a higher number of nonsynonymous mutations (**Figure 9A**). Subsequently, after searching for high-frequency mutated genes, we found four significant mutations in the low ROSig group: FBXW7, HUWE1, LYST, and TET3. FLG was a significantly mutated gene in the high ROSig group (**Figure 9B**). A detailed mutation landscape of high-frequency mutated genes was shown by waterfall plots (**Figure 9C**). We then summarized the CNV profiles of different ROSig groups, and the results showed that there were more CNV events in the low ROSig group. HSF1, in particular, underwent more amplification in the low ROSig group (**Figure 9D**). In addition, the overall chromosome amplification number and the number of deletion segments were also significantly and negatively correlated with ROSig, and both were upregulated in the low ROSig group (**Figures 9E, F**).

### Assessment of the clinical application potential of ROSig

**Figure 10:**
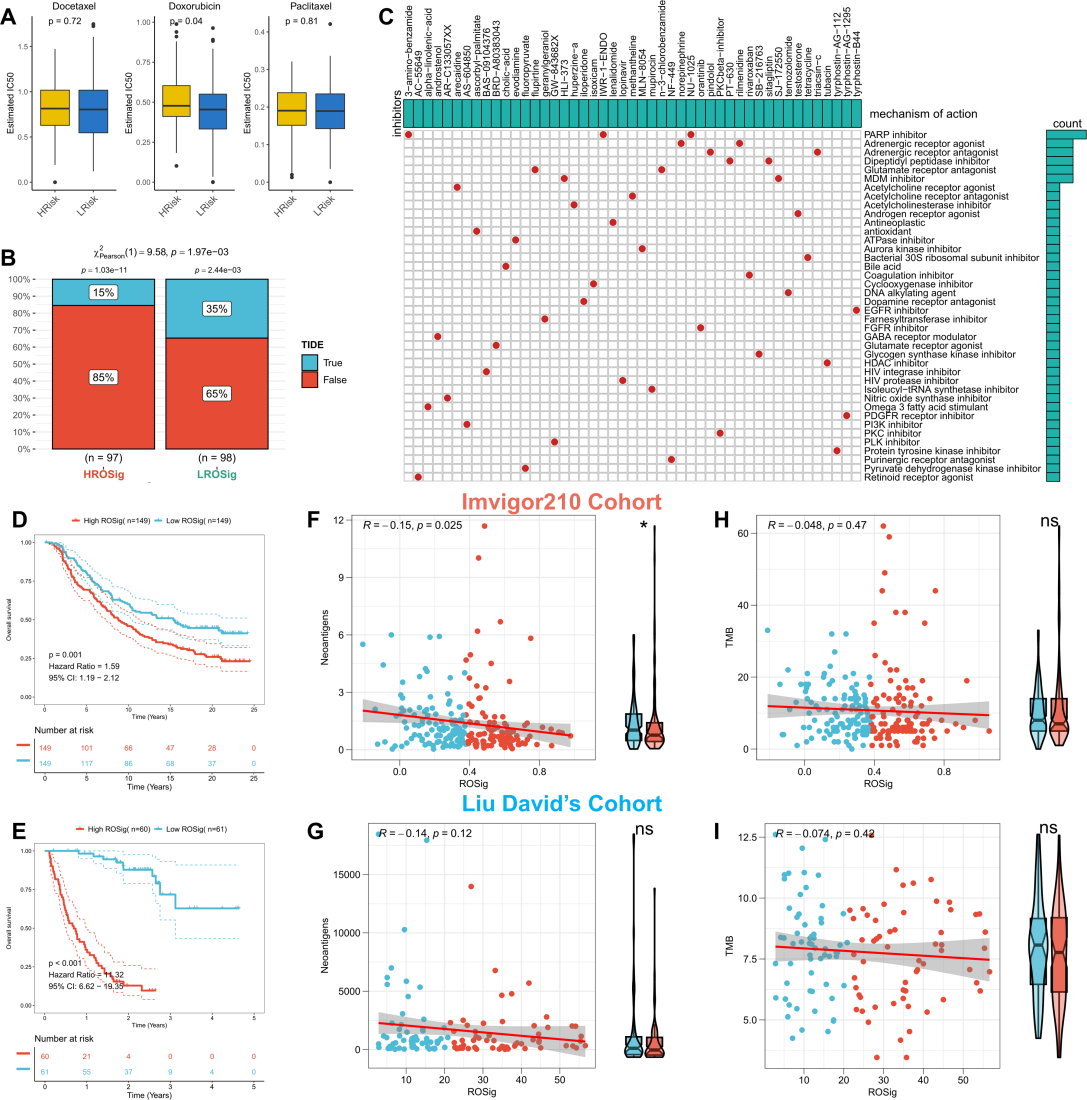
Assessing the potential of ROSig for clinical applications. **(A)** Box plots display the predicted IC50 values for three first-line drugs of TNBC in the high- and low-ROSig groups in the TCGA cohort. **(B)** Response rates to immunotherapy in different ROSig groups based on TIDE predictions in the TCGA cohort. **(C)** Forty-seven potential small molecule drugs targeting ROSig and their targeting pathways based on the Cmap database. KM survival curves for patients in the high- and low-ROSig subgroups in **(D)** Imvigor210 and **(E)** Liu David’s cohort. Box plots and scatter plots show the correlation between ROSig and neoantigens in **(F)** Imvigor210 and **(G)** Liu David’s cohort. Box plots and scatter plots show the correlation between ROSig and TMB in **(H)** Imvigor210 and **(I)** Liu David’s cohort. *P<0.05.

We first evaluated the sensitivity of different ROSig groups to three first-line TNBC chemotherapeutic agents (docetaxel, doxorubicin, and paclitaxel). The results showed no significant difference in the sensitivity of different ROSig groups to docetaxel and paclitaxel, but the low ROSig group was more sensitive to doxorubicin (**Figure 10A**). We also confirmed this finding in the validation cohort-Metabric (**Figure S1A**). Subsequently, the TIDE algorithm showed that more patients in the low ROSig group may benefit from immunotherapy (**Figure 10B**), a result that is also supported in the validation set (**Figure S1B**). In addition, 47 small molecule compounds potentially targeting ROSig were identified through the Cmap database, acting on 40 different signaling pathways (**Figure 10C**). Subsequently, we generated ROSig in two immunotherapy cohorts (Imvigor210 and Liu David’s) using the “predict” function. Survival analysis showed that patients with low ROSig showed better survival in both cohorts (**Figures 10D, E**). In addition, we analyzed the association of ROSig with TMB and neoantigens in both cohorts. The results showed a significant negative association between ROSig and neoantigens and increased neoantigens in low ROSig (**Figure 10F**). However, this was not observed in Liu David’s cohort (**Figure 10G**). In both cohorts, ROSig was not significantly correlated with TMB (**Figures 10H, I**).

### Pancancer landscape of ROSig

**Figure 11:**
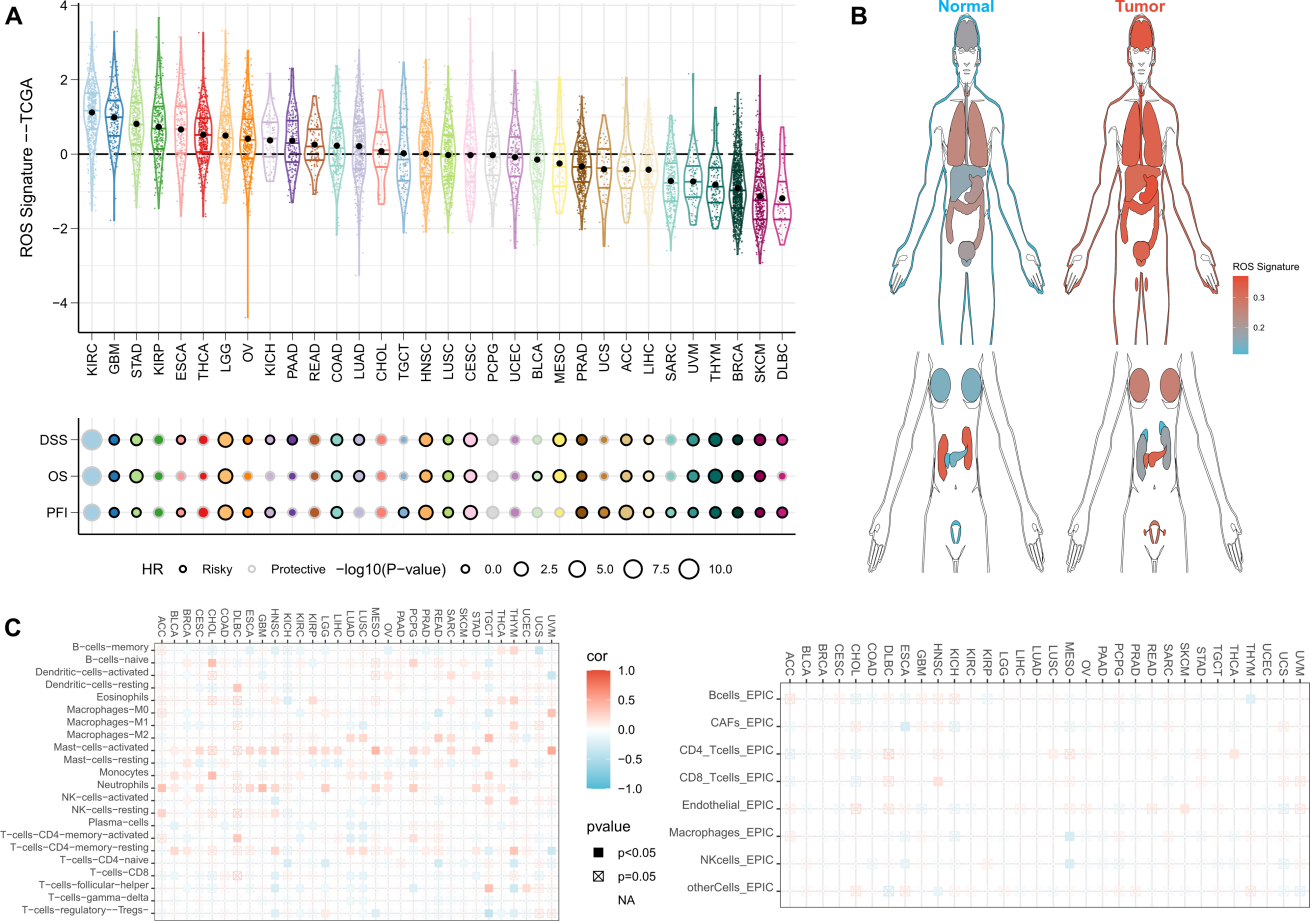
Pancancer application potential of ROSig. **(A)** Density and univariate Cox regression analysis of ROSig in 32 solid tumors. **(B)** Differentiation of ROSig in normal and cancerous organs or tissues. **(C)** The correlation between ROSig and immune cell infiltration in 32 solid tumors.

Finally, we sought to assess whether ROSig can be generalized to all types of solid tumors. We first observed that ROSig was enriched in renal clear cell carcinoma and glioma and that ROSig could serve as an accurate and robust risk factor in most types of solid tumors (**Figure 11A**). We also compared ROSig differences between normal and cancerous organs. The results showed that most organs caused an increase in ROSig after carcinogenesis, except for the kidney and pancreas (**Figure 11B**). Finally, we evaluated the association of ROSig with immune cell infiltration from a pancancer perspective and showed that ROSig was significantly positively associated with M2 macrophages in most cancer types. In particular, low levels of ROSig predicted high levels of effector cell infiltration (including M1 macrophages, T cells, and NK cells) in patients with lung cancer (**Figure 11C**).

## Discussion

BC has emerged as a major tumor type that affects women’s health (1, 2). As the most heterogeneous and aggressive molecular subtype, patients with TNBC often have difficulty responding to conventional therapies and pose a difficult challenge for targeted therapies (3). Fortunately, rapid advances in transcriptomics and single-cell genomics have provided powerful instruments for research in the field of precision medicine, and there is hope that we can find proven biomarkers to assist in prognosis and treatment decisions for TNBC patients. With the increased understanding of ROS, it is now believed that modulating ROS levels not only enhances chemotherapy sensitivity and induces apoptosis in tumor cells but also modulates immune cell activity to generate stronger antitumor immunity (11). Here, we sought to explore the key regulators of ROS in TNBC as effective biomarkers through a systematic multiomics study.

In this study, we systematically searched the transcriptional profile of TCGA-TNBC and identified 24 potential ROS regulators. We noted that all ROS regulators except PARP1 were risk factors. Interestingly, the frequency of PARP1 mutations as well as segmental amplification events were the most common. It was concluded that PARP1 has prognostic value in a variety of solid tumors and is involved in maintaining the stability of genomic genetic material (34). Thus, phenotypic alterations of PARP1 due to mutations and amplifications may be a factor in TNBC heterogeneity and poorer prognosis. Advances in machine learning provide effective new tools for diagnosis, prognosis, and treatment in clinical oncology (35). We subsequently developed ROSig for individual patient risk stratification via an integrated machine-learning pipeline. We confirmed that ROSig is a robust prognostic indicator of OS in TNBC patients with excellent predictive performance in different TNBC cohorts. More convincingly, we compared the prognostic efficacy of ROSig with 31 published molecular signatures and found that ROSig has leading predictive accuracy. In addition, we also confirmed through CCK8 that the core ROS regulator HSF1 plays a protumor proliferation role in BC cell lines.

ROS have clear regulatory effects on a variety of pro-tumor signals, and manipulation of ROS levels in tumor tissues is expected to be a novel option for cancer treatment. Therefore, we subsequently resolved the differences in biological pathways between different ROSig subgroups from a single-cell perspective and a bulk perspective. Overall, patients in the high ROSig group had less crosstalk between cells, whereas patients in the low ROSig group had abundant communication between tumor cells and cells in the microenvironment. Specifically, the MIF and MAPK pathways were more active in the high ROSig group. In contrast, most antitumor immune signaling pathways (e.g., TNF, CXCL, IL16, and IFN-γ) were active in the low ROSig group. In addition, functional enrichment analysis also confirmed that patients in the high ROSig group were mainly enriched in cell cycle-related pathways. Patients with low ROSig were more enriched in immune-related pathways. ROS induce more antigenic stimuli at appropriate levels to stimulate antitumor immunity (36, 37), and more antitumor immune signals undoubtedly enhance tumor killing by effector immune cells (38, 39). Therefore, we hypothesize that the good prognosis of patients with low ROSig may be due to an appropriate “ROS-immune” balance that allows for an enhanced antitumor immune response. This may be a new inspiration for targeting ROS as an adjunct to immunotherapy.

To explore how ROSig characterizes the different immune microenvironments, we then evaluated the microenvironmental composition of patients in different ROSig groups in detail. We found a significant increase in tumor purity, stromal score and M2 macrophage abundance in high ROSig. Previous studies have demonstrated the suppressive effect of M2 macrophages on antitumor immune responses, and abundant tumor cells may also secrete more suppressive cytokines to promote immune escape (40, 41). This ultimately leads to high ROSig corresponding to more heterogeneity and poorer prognosis of TNBC patients. In contrast, more checkpoint expression and neoantigens were present in the low ROSig group, which may promote the response of immune cells to checkpoint inhibitors (35). Therefore, we hypothesize that patients in the low ROSig group are more suitable for immunotherapy.

Alterations in the genetic material of the genome have a huge impact on the function of proteins and ultimately cause phenotypic changes. In addition, tumor mutations may generate more specific antigenic peptides to enhance immunotherapy sensitivity. Therefore, we subsequently analyzed the differences in genomic variants in patients from different ROSig groups. Surprisingly, patients with low ROSig had a higher tumor mutation load and significantly higher amplified and deletion segments on chromosomes, which are possible markers for the benefit of immunotherapy in clinical practice. In particular, significant FLG mutations were found in the high ROSig group, and studies suggest that loss of FLG function due to mutations may increase the risk of basal cell carcinoma, which may also be a mechanism for the worse prognosis of patients in the high ROSig group.

Finally, we evaluated the potential of ROSig for clinical application from multiple perspectives. First, TNBC patients with low ROSig had lower IC50 values for doxorubicin, suggesting a clinical search for TNBC patients suitable for doxorubicin regimens based on ROSig levels. In addition, the TIDE algorithm confirmed the presence of a greater immunotherapy response in the low ROSig patient group. To validate the sensitivity of immunotherapy, we generated ROSig in the immunotherapy IMvigor210 cohort and Liu David’s cohort and demonstrated that ROSig is an unfavorable prognostic factor for OS. However, we did not find a potential correlation between ROSig and tumor mutations and neoantigens. More studies are needed to elucidate how different levels of ROS affect sensitivity to immunotherapy.

ROSig has a surprisingly promising clinical application and can be detected in actual clinical practice by simple PCR to generate ROSig. Although the clinical application of ROSig in TNBC is exciting, we should also acknowledge some limitations of the study. First, the final model containing ROS genes should be further reduced to minimize financial expenses and facilitate rapid detection. Second, our analysis and predictions are based on retrospective data, and further multicenter real-world studies are needed to confirm the reliability of the model. Finally, the dataset only records a portion of the genomic data, and the actual genomic dynamic changes need more assays to assess, and our study may have overlooked some potential crosstalk and targets.

## Conclusion

In summary, we systematically evaluated potential ROS regulators in TNBC and developed a stable and efficient ROSig based on large-scale transcriptomic data and a well-established machine-learning pipeline to assist in risk stratification and treatment decisions for TNBC patients. This ROSig also allows a simple assessment of TNBC heterogeneity in terms of biological function, immune microenvironment, and genomic variation.

## Data availability statement

The original contributions presented in the study are included in the article/**Supplementary Material**. Further inquiries can be directed to the corresponding authors.

## Author contributions

Designing and supporting this study: CW. Drafting and completing the manuscript: JL. Collecting and analyzing the data: YL. Checking the quality of the manuscript and revising manuscript: XZ. Data Interpretation: YL and GH. All authors are informed of and have agreed to the final version of the manuscript.

## Supplementary material

Supplementary Figure 1 Validation of ROSig-based treatment prediction. **(A)** Box plots displayed the predicted IC50 values for three first-line drugs of TNBC in high- and low-ROSig groups in the Metabric cohort. **(B)** Response rates to immunotherapy in different ROSig groups based on TIDE predictions in the Metabric cohort.
