# Supplementary figures and images for: Integrating machine learning algorithms to systematically assess reactive oxygen species levels to aid prognosis and novel treatments for triple -negative breast cancer patients
Source: Front Immunol. 2023 Jun 19;14:1196054. doi: 10.3389/fimmu.2023.1196054 (PMC10315494; doi:10.3389/fimmu.2023.1196054)

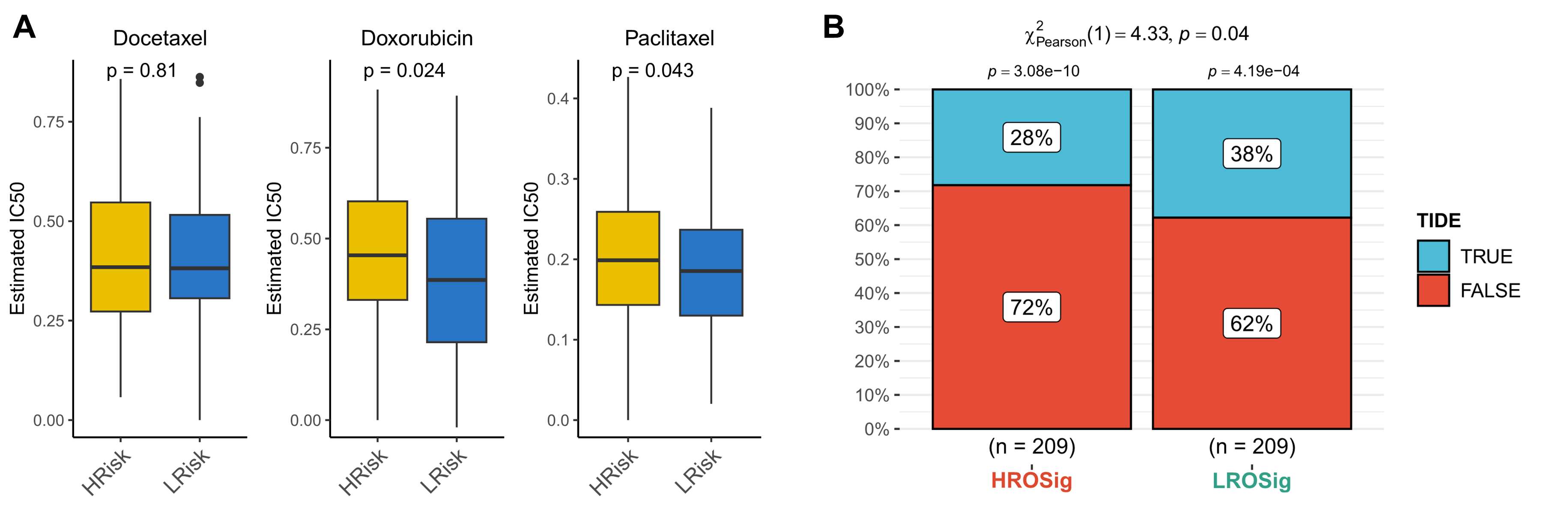

Supplement: Supplementary Figure 1 — Validation of ROSig-based treatment prediction. (A) Box plots displayed the predicted IC50 values for three first-line drugs of TNBC in high- and low-ROSig groups in the Metabric cohort. (B) Response rates to immunotherapy in different ROSig groups based on TIDE predictions in the Metabric cohort. [file Image_1.tif]
